# Exploiting the Biosynthetic Potency of Taxol from Fungal Endophytes of Conifers Plants; Genome Mining and Metabolic Manipulation

**DOI:** 10.3390/molecules25133000

**Published:** 2020-06-30

**Authors:** Ashraf S.A. El-Sayed, Manal T. El-Sayed, Amgad M. Rady, Nabila Zein, Gamal Enan, Ahmed Shindia, Sara El-Hefnawy, Mahmoud Sitohy, Basel Sitohy

**Affiliations:** 1 Enzymology and Fungal Biotechnology Lab (EFBL), Botany and Microbiology Department, Faculty of Science, Zagazig University, Zagazig 44519, Egypt; tawfeek.manal@gmail.com (M.T.E.-S.); genan11@gmail.com (G.E.); shindia21@gmail.com (A.S.); saraelhefnawy11@gmail.com (S.E.-H.); 2 Faculty of Biotechnology, October University for Modern Sciences and Arts, Cairo 12566, Egypt; amrady@msa.eun.eg; 3 Chemistry Department, Faculty of Science, Zagazig University, Zagazig 44519, Egypt; dr.nabila.zein@gmail.com; 4 Biochemistry Department, Faculty of Agriculture, Zagazig University, Zagazig 44519, Egypt; mzsitohy@hotmail.com; 5 Department of Clinical Microbiology, Infection and Immunology, Umeå University, SE-90185 Umeå, Sweden; 6 Department of Radiation Sciences, Oncology, Umeå University, SE-90185 Umeå, Sweden

**Keywords:** taxaceae, podocarpaceae, taxol biosynthesis, elicitors, fungal endophytes, genome mining

## Abstract

Endophytic fungi have been considered as a repertoire for bioactive secondary metabolites with potential application in medicine, agriculture and food industry. The biosynthetic pathways by fungal endophytes raise the argument of acquisition of these machineries of such complex metabolites from the plant host. Diterpenoids “Taxol” is the most effective anticancer drug with highest annual sale, since its discovery in 1970 from the Pacific yew tree, *Taxus brevifolia*. However, the lower yield of Taxol from this natural source (bark of *T. brevifolia*), availability and vulnerability of this plant to unpredicted fluctuation with the ecological and environmental conditions are the challenges. Endophytic fungi from *Taxus* spp. opened a new avenue for industrial Taxol production due to their fast growth, cost effectiveness, independence on climatic changes, feasibility of genetic manipulation. However, the anticipation of endophytic fungi for industrial Taxol production has been challenged by the loss of its productivity, due to the metabolic reprograming of cells, downregulating the expression of its encoding genes with subculturing and storage. Thus, the objectives of this review were to (1) Nominate the endophytic fungal isolates with the Taxol producing potency from *Taxaceae* and *Podocarpaceae*; (2) Emphasize the different approaches such as molecular manipulation, cultural optimization, co-cultivation for enhancing the Taxol productivities; (3) Accentuate the genome mining of the rate-limiting enzymes for rapid screening the Taxol biosynthetic machinery; (4) Triggering the silenced rate-limiting genes and transcriptional factors to activates the biosynthetic gene cluster of Taxol.

## 1. Introduction

Plants produce a variety of secondary metabolites, that indirectly participate on their physiological growth, regulating the interactions between plants and their environment, through various functions such as defense against pests, herbivores and phytopathogens [1]. For humans, these secondary metabolites could be used as bioactive compounds, pharmaceuticals of anti-microbial, antioxidant and anticancer activity [2]. Secondary metabolites include terpenoids, phenolic compounds, flavonoids, sulfur and nitrogen-containing compounds [3]. Approximately 50% of the most effective drugs are derived from natural products [4]. Plant terpenoids such as Taxol, sterols, gibberellins and carotenoids are implemented in commercial and pharmaceutical industries. Taxol has been reputed as the most effective anticancer drug with more than 15 billion dollars annual sale by the pharmaceutical industry [5]. Taxol is one of the most clinically valuable terpenoids since its discovery in 1970 from the Pacific yew tree, *Taxus brevifolia* [6]. It has been recognized as blockbuster anticancer compound, the most successful broad range drug against proliferation of numerous cancers such as breast, ovarian and AIDS-related sarcoma; it has been approved by Food and Drug Administration (FDA) in 1994 [7]. Taxol has a unique mode of action by binding to β-tubulin, promoting the microtubule assembly and disrupting the mitotic division of the target cells [8].

The current approaches for Taxol production are; 1- Natural sources from the bark of *T. brevifolia* (most productive source), however, the yield of taxol based on this approach was ranged from 0.001–0.05%, thus for producing of one gram purified Taxol it need about 8–10 kg of plant bark, which collected from about 4 to 5 plants [9]. However, the scarce availability and higher vulnerability of this plant to unpredicted fluctuation with the ecological and environmental conditions [10] are the challenges for this source [3,11,12,13]. 2-Semisynthetic process via 10-decaetylbaccatin III intermediate from the needles of *T. baccata* is the current approach for taxol production [14,15], however, the lower yield of this intermediate, selectivity over unwanted byproducts, heterogeneity, reproducibility, in addition to the epigenetic and mutational changes of *T. baccata* are the current hurdles [16,17]. 3-Endophytic fungi from *Taxus* spp. opened a new avenue for industrial Taxol production due to their fast growth, cost effectiveness, independence on climatic changes and feasibility for genetic manipulation [18,19]. *Taxomyces andreanae* was the first reported Taxol producer endophyte from *Taxus* spp. [18]. More than 150 fungal endophytes were identified from *T. baccata* and about 10% of this population has the potentiality to produce Taxol [20]. However, the anticipation of endophytic fungi for industrial Taxol production has been challenged by the loss of Taxol productivity with multiple subculturing [21,22,23,24,25,26,27,28]. Downregulation of the expression of Taxol genes upon subculturing of the endophytic fungi was reported frequently [29]. Thus, searching for novel fungal saprophytes having a stable molecular machinery system for conceivable production of Taxol independently is the ultimate objective for biotechnologists [22,30].

## 2. Chronology of Taxol, its Derivatives as Antiproliferative Drug

**Figure 1:**
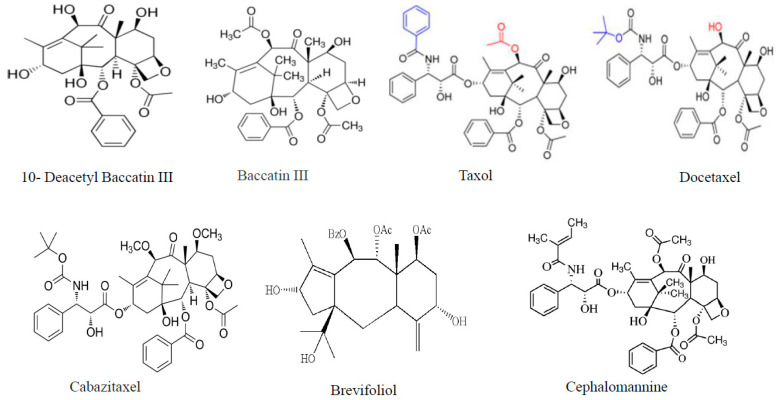
Chemical structure of Taxol and other Taxoid compounds.

Following the National Cancer Institute (NCI) program of searching for novel anticancer compounds in 1960, Taxol and camptothecin were the first explored compounds with strong antiproliferative activity [31]. Taxol has been clinically investigated by NCI clinical trials in the following phases: Phase I (breast, liver, ovarian epithelial, lymphoma and childhood leukemia), Phase II (colon, head and neck, renal cell, prostate, small cell lung cancers, esophageal cancer and melanoma) and Phase III (metastatic breast cancer and ovarian epithelial cancer). In 1992/ 1994, the Food and Drug Administration (FDA) approved Taxol as an effective drug against ovarian and breast cancer, respectively. Thus, Taxol was considered as one of the most important additions to the chemotherapeutic field in the late 20th century. The chronology of Taxol discovery, development and its clinical trials has been documented [23,30,32,33,34]. In the late 1980s, diterpenoid Taxane or Taxoid derivatives, Taxol and Docetaxel were introduced and proved to be effective in treatment of a variety of solid tumors including ovarian, lung, breast and bladder cancers [35]. Also, paclitaxel semisynthetically supplied from precursor, 10-deacetylbaccatin III that derived from European yew *Taxus baccata.* Docetaxel—also named Taxotere—is semi synthetically from 10-deacetylbaccatin III. It is reported that Docetaxel is more soluble than paclitaxel. Chemically, two drugs, Paclitaxel and Docetaxel composed of a taxane ring linked to an ester bond at the C-13 position (Figure 1). Docetaxel (Taxotere) and Paclitaxel (Taxol) considered as the standard chemotherapies for patients with metastatic breast cancer [35,36]. Cabazitaxel is the second taxane derivative that has been discovered to overcome docetaxel resistance and to improve overall survival in metastatic castration-resistant prostate cancer patients pretreated with Docetaxel [37]. A novel diterpene taxane, brevifoliol (Figure 1) has been reported to be found in Needles of *T. brevifolia* [38], with a cytotoxic activity against breast, liver, oral and colon cancer cell lines. Cephalomannine has been reported to be isolated from *Taxus wallichiana* [39] and from other yew species [40]. Cephalomannine structure differ from that of paclitaxel only in its C-13 side chain: paclitaxel has an N-benzoyl group in its C-13 side chain, while cephalomannine has N-tigloyl group. The chemicals structures of Taxol and some of its related Taxoid compounds were illustrated in Figure 1.

Taxol is a crystalline powder with white to off-white color, melting temperature 216 °C, with benzene rings as the hydrophobic structures rendering it highly lipophilic. It has poor solubility in water (<1 μg/mL) [41], can soluble in organic solvents such as methanol, tertiary butanol, DMSO, methylene chloride and acetonitrile. Clinically, due to low oral bioavailability (<2%) and poor water solubility of Taxol, the commercial products of paclitaxel combine with Cremophor EL (CrEL) (Castor Oil), a formulating vehicle used for poorly water-soluble drugs and administered by intravenous injection [42]. However, addition of Cremophor EL to paclitaxel has side effects such as cardiotoxicity, neurotoxicity and nephrotoxicity that reduces the drug efficacy limits its use [42]. Several pharmaceutical vehicles have been proposed to overcome this problem such as polymer nanoparticles [43], solid lipid nanospheres [44], nano-emulsions [45], paclitaxel-loaded nanosponges [46], paclitaxel stabilized micelles [47], pluronic /LHR mixed polymeric micelles [48] and paclitaxel microemulsions [49].

## 3. Taxol Biosynthesis

Terpenoids are biosynthesized from its precursor isopentenyl Pyrophosphate (IPP) that inter-converts into dimethylallyl pyrophosphate (DMAPP) by Isomerase through protonation process followed by deprotonation (isoprene units, C5). Isoprenoids are biosynthesized in all organisms through the mevalonic acid (MVA) pathway or methylerythritol 4-phosphate (MEP) pathway. It is reported that plants can use both MVA pathway and MEP pathway for synthesis of terpenoids. Mevalonic acid pathway was discovered at 1950s in fungi, yeast, archaebacteria and animals, while, MEP pathway was discovered in most of eubacteria, parasites and plants [50,51].

**Figure 2:**
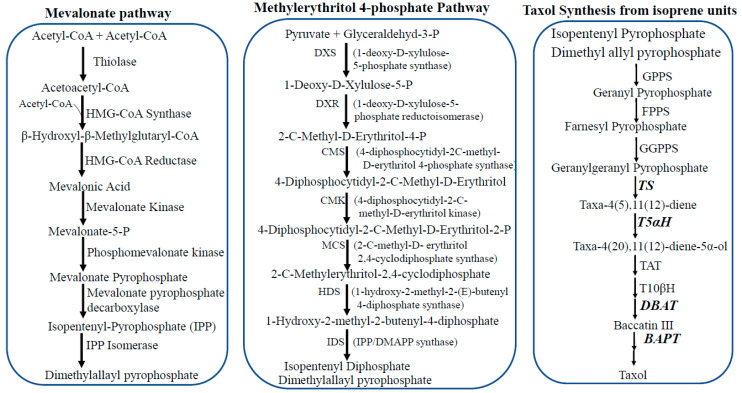
Biosynthetic pathways of isoprene units such as isopentenyl pyrophosphate (IPP) and dimethylallyl pyrophosphate (DMAPP). Mevalonate pathway, Methylerythritol 4-phosphate and Taxol biosynthesis from the isoprene units. Biosynthesis of terpenoids in fungi. Mevalonate and isoprenoids pathways are the main sources of isopentenyl pyrophosphate (IPP) and dimethyl allyl pyrophosphate (DMAPP) (isoprene units, C5). Farnesyl pyrophosphate (FPP) is the precursors of diterpenoids (Taxol, C20) by condensation reactions catalyzed by geranylgeranyl pyrophosphate synthase (GGPPS). Abbreviations of enzymes; GPPS, geranyl pyrophosphate synthase; FPPS, Farnesyl pyrophosphate synthase; GGPPS, geranylgeranyl pyrophosphate synthase; TS, taxa-4(5),11(12)-diene synthase; DAPT, baccatin III -13-O-(3-phenylpyropanyoyl) transferase (DAPT).

Mevalonic acid pathway “HMG-CoA reductase pathway” is the source of isopentenyl pyrophosphate and dimethylallyl pyrophosphate in cytosol of cell. Mevalonic acid is synthesized from acetyl-CoA derived from nutrients (Figure 2). In the MVA pathway, isopentenyl pyrophosphate (IPP) is biosynthesized from three molecules of acetyl CoA, two molecules of Acetyl CoA united together by thiolase giving acetoacetyl CoA with further one molecule of acetyl-CoA by hydroxyl methyl glutaryl CoA synthase giving hydroxymethyl glutaryl CoA (HMG-CoA) which undergoes reduction by hydroxymethyl glutaryl CoA reductase giving mevalonic acid. Mevalonic acid activated into mevalonic acid monophosphate and pyrophosphate by kinases. Mevalonic acid pyrophosphate is converted into isopentenyl pyrophosphate (IPP) by mevalonic acid pyrophosphate decarboxylase, then by isomerase into dimethylallyl pyrophosphate (DMAPP) [52,53].

Methylerythritol 4-phosphate (MEP) or non-mevalonate pathway (NMVA) or Deoxy-xylulose phosphate (DXP pathway) synthesizes isopentenyl pyrophosphate and dimethylallyl pyrophosphate in plastid of plant cell, independent on mevalonic acid (Figure 2). In this pathway, pyruvic acid was complexed with glyceraldehyde 3-phosphate by deoxyxylulose phosphate synthase forming deoxyxylulose 5-phosphate (DXP) which undergoes reduction forming methylerythritol 4-phosphate (MEP) which undergoes multiple steps forming Isopentenyl pyrophosphate (IPP) and its isomer dimethyallyl pyrophosphate (DMAPP) [13,21,22,23,24,25,32,34,54,55,56,57,58,59,60,61,62,63,64].

Taxol was reported to be synthesized firstly in *Taxus* from geranylgeranyl pyrophosphate (GGPP) (C20), precursor for taxadiene, which formed by coupling of isopentyl pyrophosphate (C5) and farnesyl pyrophosphate (C15), cyclization, oxygenation and substitution of several functional groups via 19 steps of chemical reactions [65,66]. Taxol biosynthesis has been summarized in Figure 2. The steps of Taxol biosynthesis started with (1) coupling of isopentenyl pyrophosphate with farnesyl pyrophosphate via geranylgeranyl pyrophosphate synthase (GGPPS) forming geranylgeranyl pyrophosphate (C20) (2) cyclization of GGPP by taxadiene synthase (TDS) forming taxa-4(5),11(12)-diene (3) taxa-4(5),11(12)-diene undergoes hydroxylation at carbon #5 via cytochrome P450 taxadiene 5α-hydroxylase (TYH5a) to form Taxa-4(20),11(12)-diene-5α-ol [67] (4) taxa-4(20), 11(12)-diene-5α-ol undergoes acylation via taxa-4(20),11(12)-diene-5α-ol-O-acetyltransfer-ase (TAT) forming taxa-4(20),11(12)-dien-5a-ylacetate [13], (5) taxa-4(20),11(12)-dien-5a-yl acetate undergoes hydroxylation by cytochrome P450 taxadiene 10b-hydroxylase (TYH10b) forming Taxa-4(20),11(12)-dien-5α-acetoxy-10β-ol which converts into 2-debenzoyl taxane through formation of oxetane and other undefined steps. (6) 2-O-debenzoyl taxane undergoes benzoylation via a taxane 2a-O-benzoyltransferase (TBT) forming 10-deacetyl baccatin III. (7) 10-deacetyl baccatin III undergoes acetylation via 10-deacetyl baccatin III-10-O-acetyltransferase (DBAT) forming baccatin III. (8) Side-chain of baccatin III attached to phenyl-propanoyl via the baccatin III 13-O-(3-amino-3-phenyl-propanoyl) transferase (BAPT) forming 3′-N-debenzoyl-2′-deoxtaxol, then undergoes benz-amidation via 3′-N-debenzoyl-2′-deoxytaxol-N-benzoyltranserase (DBTNBT) forming Taxol [9,68].

## 4. Mode of Action

Taxol is one of the most successful drugs with anticancer activity against wide range of cancers including breast, ovary, lung, AIDS-related Karposi’s carcinoma and head carcinomas [69,70]. Cancer cells proliferation occurred by increasing in number of cells as a result of cell growth and cell division. Tubulin is a globular protein found as a component in the cytoskeleton in eukaryotic cells, with important role during mitosis of cancer cell. The dynamic of microtubules that includes polymerization (growing) and depolymerization (shrinkage) is very important during separation of chromosomes of mitosis division. Two types of tubulin, α and β tubulins polymerize together to form α-β-heterodimer microtubules [71] that assemblies head-to-tail to form protofilaments as shown in Figure S1. It has been reported that microtubules living cells have 13 protofilaments assembly parallel to the microtubule axis. Microtubule has two charged ends, positive charged end (β-tubulin) that binds to kinetochore of chromosomes and negative charged end (α-tubulin) that bind to spindle pole. Drugs binds to tubulin leads to modifying the assembly properties of microtubules. Taxol is anti-proliferative drug with a unique mode of action against the cancer cell. It binds to β-tubulin by interaction with an amino-terminal region that consists of 31 amino acids in length [72], leads to preventing the depolymerization (shrinkage) of microtubule [8,73] and blocking the cell cycle [8]. In the presence of Taxol, microtubules assembled to have 12 protofilaments rather than 13 protofilaments [74].

## 5. Sources of Taxol Production

### 5.1. Natural Source

Taxol has been isolated firstly from bark of *Taxus brevifolia,* the pacific yew or Western yew, a tree of the family Taxaceae that found in the western United States [6]. Due to effective anticancer activity of Taxol, members of genus *Taxus* producing Toxoids, a class of Taxol derivatives, have been distributed in Europe, Asia, North and Central America [75]. More than three-hundreds Taxoid compounds have been isolated [38].

Natural sources from the bark of *T. brevifolia* (most productive source), the yield of taxol ranged from 0.001–0.05% [31]. Availability and vulnerability of this plant to unpredicted fluctuation with the ecological and environmental conditions are the challenges for this source. Thus, for production of 1 g of taxol, it needs about 10 kg of Taxus bark (~60 old), which is produced approximately from three trees and every cancer patient requires about 2.5 g [9]. 2-Semisynthetic process via 10-decaetylbaccatin III intermediate from the needles of *T. baccata* is the current approach for taxol production [18,19,76], however, the lower yield of this intermediate, selectivity over unwanted byproducts, heterogeneity, reproducibility, in addition to the epigenetic and mutational changes of *T. baccata* are the current hurdles [29,77]. 3-Endophytic fungi from *Taxus* spp. opened a new avenue for industrial Taxol production due to their fast growth, cost effectiveness, independence on climatic changes and feasibility for genetic manipulation [3,13,78,79]. *Taxomyces andreanae* was the first reported Taxol producer endophyte from *Taxus* spp. [80]. More than 150 fungal endophytes were identified from *T. baccata,* about 10% of this population has the potentiality to produce Taxol [81]. However, the anticipation of endophytic fungi for industrial Taxol production has been challenged by the loss of Taxol productivity with multiple subculturing [81].

#### 5.1.1. Family Taxaceae; Taxonomy and Ethnopharmacological Use

Taxaceae is a slow-growing coniferous yew family distributed mainly in the Northern Hemisphere. Taxaceae has been included on *Coniferales* based on their morphological, anatomical, chemical and chromosomal characters [82] and also based on their chloroplast DNA structural mutation [83]. Taxaceae has been included on Coniferales based on their 18S rRNA sequences of Taxus, Podocarpus, Gingko and Pinus [84]. Taxus is the most widely distributed genus in *Taxaceae* family. As a feature of distinguishing between *Taxaceae* family members, the genus Taxus was found to have microsporophyll, red aril and variable foliage that gave it extreme importance in a horticultural industry. There are several types of yew such as English Yew (*Taxus baccata*) that distributed in United States, Pacific or Western Yew (*Taxus brevifolia*), American Yew (*Taxus canadensis*), Japanese Yew (*Taxus cuspidata*) (Cope 1998). It has been reported that Taxaceae family includes either five genera *Taxus, Pseudotaxus, Austrotaxus, Torreya and Amentotaxus* [85] or only the first four of these five genera [86].

It has been reported that wood of *Taxus brevifolia* has been used by the people of British Columbia for wedges, needles, bows, digging sticks and whaling harpoons. *T. brevifolia* bark has been used by many tribes in medicine [87]. A steeped solution of *T. brevifolia* bark has been used by Coast Salish people of British Columbia to treat ailments of tuberculosis, liver, kidney stomach and digestive tracts [88]. Also, Yew wood has been used for ceremonial purposes such as red paint and fish oil [88]. In 1960s, National Cancer Institute of the United States started its program of screening natural products for anti-cancer activity including bark of *Taxus brevifolia.* That leads to discovering a potent anti-cancer drug, paclitaxel [89]. Needles of *T. brevifolia* have been reported to contain other taxane, brevifoliol, a novel diterpene [38], with potent cytotoxic activity against breast (MCF-7), liver (HepG-2), oral (KB) and colon (CaCO_2_) cancer cell lines [73]. It has been reported that other taxane derivatives such as deacetyl paclitaxel, Baccatin III and cephalomannine extracted from dried needles of several Taxaceae members [75]. Morphologically, *T. brevifolia* tree is a small to medium sized evergreen coniferous tree about 10-15 m tall with trunk that reaches about 50 cm in diameter covered by thin scaly brown bark. *T. brevifolia* leaves are flat, lanceolate shape, dark green.

#### 5.1.2. Family *Podocarpaceae*; *Taxonomy* and Ethnopharmacological Uses

Podocarpaceae is the second largest family of conifers distributed mainly in southern Hemisphere [80]. Most of *Podocarpus* plants are distributed in china, South-eastern Pacific Ocean islands, southeastern Africa and South America. *Podocarpaceae* family has 125 species classified into 19 genera [90]. *Podocarpaceae* family have seven genera namely; *Podocarpus, Phyllocladus, Dacrydium, Microcachrys, Acmopyle, Pherosphaera* and *Saxegothaea* [91]. The *Podocarpus* was subdivided according to the leaf anatomy into eight subgenera, *Afrocarpus* (*Podocarpus*), *Microcarpus, Eupodocarpus, Dacrycarpus, Sundacarpus, Nageia, Polypodiopsis* and *Stachycarpus* (https://www.conifers.org/po/).

Species of family Podocarpaceae *Nageia nagi,* was used as an herbal dietary supplement in USA. The fleshy reproductive structures (receptacles) of *Podocarpus elatus, P. totara, P. macrophyllus, Dacrydium cupressinum, Dacrycarpus dacrydioides* and *Afrocarpus falcatus* are eaten either raw or cooked [80]. The bark of some species contains 3–6% tannin and used for tanning leather [80]. *Podocarpaceae* has several medicinal benefits for humans and animals [92]. A variety of bioactive compounds—antioxidant, podocarpic acid, tatarol, various diterpenoids and flavonoids—have been extracted from receptacles and leaves of *Podocarpaceae* [92]. Some of these bioactive compounds reported to have useful biological activities and cytotoxic properties [93], antimicrobial, bacteriostatic and fungistatic compounds [92]. *Podocarpus macrophyllus* stem bark used to treat blood disorders and worms, fruits decoction used as a tonic for heart, lungs, kidneys and stomach [94]. *Podocarpus nagi* fruit has been used as carminative, pectoral and stomachic, its bark used as an antiseptic, carminative and treat fevers, asthma and coughs arsenic poisoning, skin diseases and ulcers. The bark extract of *Podocarpus gracilior* and *P. nakaii* has been used as antitumor agent [81]. Morphologically, *P. gracilior* tree reaches up to 60 feet in height. Its trunk free of branches for a considerable distance from its base, covered by brown cork. *P. gracilior* tree has willowy stem branches with long, narrow, spirally arranged opposite/ subopposite, simple, entire margin, lanceolate, needle-like leathery leaves and deep green, with blade up to 10 cm long on young trees and 6 cm long and on older specimens [95]. *P. gracilior* is the source of important phytochemicals. Leaves and twigs of *P. gracilior* contains Podolide, a norditerpene dilactone, with anti-tumor activity, antileukemic [96]. *Podocarpus gracilior* was the first species outside Taxaceae to produce the Taxol, with yield of 0.54 mg/kg [81]. This discovery supports a phylogenetic affinity between *Podocarpus* and *Taxus.*

### 5.2. Taxol-Producing Endophytic Fungi from Taxus and Podocarpus Species

Owing to the growing demand for Taxol and shortage of mature tree of *T. bervifolia*, searching for alternative sources like chemical synthesis, plant cell culture, microbial fermentation of endophytes and metabolic engineering of microorganisms raise the hope for industrial production of this drug [18]. The total global market of taxol is above $ 1 billion per year, with more predictable grossing with anticipation trials of this drug in various diseases [13]. Tissue culture technology achieves a successful development for paclitaxel production, but the long incubation time is still one of the main limiting factors with the urgent accessibility of cancer patients worldwide.

Endophytes are microorganisms which colonize the internal tissues of plants without causing any harmful effects on their host plants. Endophytic fungi exert uncountable protective effects on plants through their interactions, stimulating the production of important secondary metabolites, that could have both significant positive and negative influences on human health [97]. Some of these microorganisms produce natural products with effective uses in agriculture, medicine and industry [98]. So, endophytes considered as chemical synthesizers inside their host plants. The natural compounds isolated from these endophytes such as terpenoids, alkaloids and flavonoids having biological activity against different human pathogens and used as effective drugs against different types of diseases. So many natural compounds are reported to use as antibiotics, anticancer agents. Many endophytic fungi isolated from different medicinal plants are reported to be main source of many secondary metabolites and pharmaceutical compounds with biotechnological roles [3,11,12,13,21,22,23,24,25,26,27,28,32,34,54,55,56,57,58,59,60,61,62,63,64,78,79,97,99,100,101,102,103,104]. Fusarubin and anhydrofusarubin, isolated from the *Cladosporium* species an endophyte of *Rauwolfia serpentina*, displayed a promising antimicrobial and antitumor activities [105]. Therefore, endophytic fungi from the medicinal plants, could be a novel platform for commercial production of the bioactive metabolites, due to their feasibility of molecular and metabolic manipulation, short life span comparing to the plant hosts itself [13,33,78,97,99].

Endophytic fungi of *P. falcatus* displaying a remarkable activity against various phytopathogens [106]. Taxol has been identified from *P. gracilior* (African Fern Pine) [81] and this was the first report to identify Taxol from plants outside the family *Taxaceae*, revealing the closely taxonomic relations of *Podocarpaceae* and *Taxaceae* (Chaw et al. 1993b). However, the lower concentration of Taxol in *P. gracilior* leaves and stems (> 0.54 mg/kg dry weight tissues) preclude its further uses for commercial production [81].

Taxol was firstly isolated from endophytic fungus, *Taxomyces andreanae* [19], with Taxol yield bout 24–50 ng/L. This discovery directs the researcher’s attention, to search for novel endophytic fungi with higher potency and sustainability to produce Taxol from other plant hosts rather than *Taxus* species [107,108]. However, Taxol yield by endophytic fungi has been noticed to be very low and unstable, varying from 24 ng to 70 μg/L of culture [19]. Also, Taxol yield from strain of *Pestalotiopsis microspora* has been reported to be genetically unstable, varying from 50 to 1487 ng/L [109]. List of endophytic fungi isolated from different plant hosts and their Taxol yield was summarized in Table 1. Taxol produced by endophytic fungi has been expanded for treatment of metastatic ovarian carcinoma, breast, non-small-cell lung cancers and second-line treatment of AIDS-related Kaposi’s sarcoma [110].

Fungal endophytes from different *Taxus* spp. were reported to have a strong correlation with Taxol productivity, plant growth promoters and other secondary metabolites [127]. Taxol biosynthesis by *Taxus* sp. was significantly increased by coculturing with *Fusarium mairei* or its purified oligosaccharides [107] that was explained by the microbial cross talking due to activation of some cryptic genes. Endophyte taxol producer *Paraconiothyrium* sp. induced the expression of taxol biosynthetic gene in *Taxus* sp. that makes the plant more resistant to specific pathogens. Strikingly, the fungus has a potential selectivity to inhibit the host plant pathogenic fungi. Taxol-biosynthetic genes in *Paraconiothyrium* sp. were induced (eightfold higher) by both non-taxol-producing endophytes as *Alternaria* sp. and *Phomopsis* from the same host plant “Taxus” [128]. Notably, all the endophytes producing taxol are naturally resistant to taxol which has been shown as a powerful fungicide to plethora of phytopathogenic fungi [25,28,34,54,57,64,99,103]. *Taxomyces andreanae* was the first isolated Taxol-producing endophyte from *Taxus* spp., raising the hope for production of taxol via fermentation process, because of its fast growth on simple cultural media, possibility for growing on bulk fermenters, resistance to shearing and feasibility for genetic manipulation [18]. Acetate and phenylalanine were the precursors of fungal taxol and its yield was dramatically increased in presence of sterol biosynthesis inhibitors as tebuconazole and triadimefon [34]. Several fungal endophytes belonging to *Ascomycetes* and *Deutromycetes* have been recognized as promising paclitaxel producers, among these genera *Pestalotia, Pestalotiopsis, Sporomia, Trichothecium, Tubercularia, Alternaria, Pithomyces, Monochaetia, Penicillium* and *Fusarium* [129].

Interestingly, 150 fungal isolates were identified as endophytes from *T. baccata*, more than 10% of this population have the ability to produce taxol such as *Alternaria, Beauveria, Epicoccum, Fusarium, Geotrichum, Phoma, Nodulasporium, Phomopsis* and *Fusarium solani* [20]. Taxadiene synthase (TS) has been used as molecular marker for screening of Taxol-producing fungi from *T. chinensis*. Among the 38 endophytic fungi, 12 isolates were reported as TS positive but only 3 isolates gave a detectable yield of taxol. Additionally, 10-deacetylbaccatin III-10-O-acetyl transferase (DBAT) and phenyl propanoyl side chain CoA acetyltransferase (BAPT) are more diagnostic markers for taxol biosynthesis [114]. Medicinal plants which contain intrinsic substances in its tissues with therapeutic importance against different types of diseases or precursor for different drugs, thus, it become main source of drugs for world’s population where phytochemical products from plants constitute about 25% of prescribed medicines [130]. The list of endophytic Taxol- producing fungi isolated from *Taxaceae* and *Podocarpaceae*, other plants species, in addition to rhizospheric fungi are shown on Table 1.

## 6. Maximizing Taxol Bio-Production Strategies

### 6.1. Molecular Manipulation of the Microbial Strain

Strain improvement by increasing the rate of microbial growth and fermentation yield via generating new genetic characters by mutation is the common approach for maximizing the yield of Taxol by microbes (Table 2). Microbial engineering is the reliable platform for large scale production of terpenoids in a cost-effective fermentation process, independent on climate changes and cultivation risks. Several metabolic approaches have been implemented based on increasing the influx of IPP via mevalonate pathway [28]. Taxol biosynthesis is controlled by 19 enzymatic steps that are part of the terpenoid pathway; thus, several trials were conducted to increase the yield of taxol via overexpression of these enzymes in yeast or *Escherichia coli.* Overexpression of pyruvate dehydrogenase, acetaldehyde dehydrogenase and acetyl-CoA synthetase for increasing the supply for acetyl-CoA has a significant positive effect on the yield of terpenoids synthesis in *S. cerevisiae* [131]. Recently, engineering of mevalonate production by introducing mevalonate decarboxylase B mevalonate bypass, reducing the total number of ATP from three to one and the number of enzymes from seven in original pathway into four, has a strong great effect on the taxol yield [132]. Overexpression of taxadiene synthase, HMG-CoA synthase and reductase and GGPP synthase in *S. cerevisiae* has positively effect on the taxadiene yield that was increased by about 40-fold [29]. Due to the complexity of enzymatic system for terpenoid synthesis, there are multiple methods for enhancing the yield of taxol such as blocking/ silencing the competitive pathway, overexpression of the key genes and increasing the feeding precursors [133]. Metabolic engineering of microbial cells to enhance the flux of precursor pools of IPP and DMAPP via overexpression of mevalonate and isoprenoid pathway rate-limiting enzymes has been recognized as a reliable tool for enhancing the yield of terpenoids. 

Mutagenesis of mycelium is difficult to manage and optimize the conditions for mutagenesis in the case of spores, while mutagenesis on protoplasts does not have these obstacles [117]. Further, the lack of a cell wall makes protoplasts more sensitive and thus protoplast mutagenesis is a very beneficial strategy for yield enhancement in endophytic fungi. Recently, optimization of the conditions for protoplast preparation and regeneration under control of pH, temperature, time, enzyme combination, osmotic stabilizers, pretreatment, regeneration medium have an effect on the success rate [113,117]. Protoplast fusion is the current technology that has been utilized to enhance the Taxol yield for most of endophytes [121]. Secondary metabolite gene cluster amplification, gene disruption and cloning of regulatory genes are the powerful techniques to boost the Taxol yield by microbes [136]. A method for PEG-mediated transformation of Taxol producing endophytic fungus *Ozonium* sp. was established [137,138]. *Agrobacterium tumefaciens* mediated transformation (ATMT) method for Taxol-producing fungus, *Ozonium* sp. was used to setup a desirable transformation efficiency compared to the common PEG-mediated protoplast transformation [137,139].

### 6.2. Bioprocess Optimization Strategy for Taxol Production

Manipulation, culture condition optimization, elicitor/inhibitor addition and precursor feeding are the most adopted approaches used for enhancing the Taxol yield by fungal cultures. Optimization of the fermentation parameters can be achieved either through altering one parameter at a time or via varying elements and their interactions at the same time using the response surface methodology optimization process [140]. By optimizing and controlling fermentation parameters with consistent product quality and quantity independent on the environmental variations, it can be developed for industrial scale-up of Taxol production [141]. Elicitors are signaling molecules that trigger the formation of secondary metabolites in cell cultures [34]. Elicitors are labeled as biotic and abiotic based on their identity, elicitation of microbial system becomes a common platform for enhancement of their secondary metabolites production, strategy for sustainable metabolite production in fungal fermentations at large scale. In addition, use of inhibitors to divert the metabolic flux toward the biosynthesis of Taxol has been documented frequently [28]. Specifically, in case of Taxol producing endophytes, production of quantities of ergosterol are usually observed, usage of sterol biosynthesis inhibitors to divert the geranyl-geranyl pyrophosphate pool towards Taxol biosynthesis enhancing the Taxol yield up to 50 folds [107]. The yield of Taxol produced by *Aspergillus flavipes* was elevated up to 5 folds with sterol biosynthesis inhibitor (fluconazole) addition [34]. Examples of elicitor/inhibitor addition for enhancement Taxol yields fungi are listed in Table 2.

Precursor feeding is a strategy of exogenously presenting a biosynthetic precursor or different intermediates in the biosynthetic pathway to the culture medium to enhance the desired product yield [3,11,12,13,21,22,23,24,25,26,27,28,32,33,34,54,55,56,57,58,59,64,78,79,97,99,100,101,102,103,104]. It is a broadly used approach in plant cell/ tissue cultivation and being explored in the fermentation of endophytic fungi. Precursor feeding approaches have additionally represented a main role in the elucidation of biosynthetic pathways of Taxol [19].

## 7. Co-Cultivation and Mixed Fermentation

Co-cultivation of two or extra organisms for eliciting Taxol biosynthesis is a common strategy to stabilize the productive potency of the fungal cultures, via triggering the silenced biosynthetic genes clusters in fungi (reviewed by [13]. Co-culturing approach exhibited a positive effect on utilizing ‘auxiliary’ traces via the presence of bioactive molecules. The yield of Taxol has been accelerated by three times via co-culturing of fungus with the bark of plant [134]. Additionally, the biosynthesis of Taxol was increased up to 38 folds by co-culturing of *Fusarium* sp. with *Taxus* suspension cells [107]. The average yield of Taxol via *A. terreus* was elevated up to 2.5 folds and 2.1 folds upon addition of surface sterilized leaves and corks of *P. gracilior*, respectively [103].

## 8. Genome Mining

Endophytic fungi live in the tissues of plants without causing any symptoms of disease [28,33,60,97,142], with a mutualistic relationship with the plant, providing the plant host the proper defense mechanism against the phytopathogens by secreting bioactive metabolites and phytohormones. Thus, endophytic fungi have been considered as a repertoire for bioactive secondary metabolites with potential application in medicine, agriculture and food industry [143]. Production of novel metabolites by fungal endophytes raise the questions about the acquisition of the biosynthetic machineries of such complex metabolites by these organisms from the plant host, as concluded from the similar biosynthetic pathway and genes encoding enzymes catalyzing various steps with the plant host [144]. Each plant species is known to harbor many endophytic fungi, however, few of them were recognized as bioactive metabolite producers, therefore, to identify the potential isolates capable of producing a particular compound, there is a need to screen all the isolated endophytes, which are uncountable and cumbersome process [121,145]. For screening of the organism of interest with the target metabolite, there are two main approaches (1) traditional methods and (2) molecular methods.

Traditionally approaches mainly implemented starting from isolation of endophytes, culturing on desired media, biological activity-guided assessment, extraction, purification, identification and chromatographic and spectroscopic characterization of the bioactive molecules [13,33,61,78,97,146]. Although, such techniques are powerful tools for the identification and characterization of new desired metabolites, however, the isolation and screening for the target fungal isolate is a very laborious process, time- consuming, with long-step processing, requiring a lot of facilities. Thus, there is a need for developing an efficient procedure for the screening of large number of isolated endophytic fungi to identify the strains capable of producing specific novel pharmaceutically important compounds.

Molecular approaches for screening of bioactive compounds from endophytic fungi has been used as a sophisticated strategy especially with the availability of enormous genetic data from the genomic and metabolomic analyses. Genome mining by exploring the whole-genome sequence to identify biosynthetic pathways “gene cluster” of novel metabolites is the prospective approach to overcome the long-term processing of traditional screening methods [144]. Among the filamentous fungi, the genus *Aspergillus* is known to produce a variety of natural products with broad spectrum biological activity. Genome sequencing of various species of *Aspergillus* displaying a multiple number of gene clusters, that encodes numerous secondary metabolites still to be obscure. These gene clusters (GC) are physically co-localized genes participating on the same metabolic pathway, producing novel compounds, these clusters are usually evolved in the fungal lineage in response to specific ecological desires for production of specific secondary metabolites. Genome mining for bioactive secondary metabolites is greatly facilitated by the cutting-edge high-throughput genome and transcriptome sequencing approaches [123].

These gene clusters usually encode a complex enzymatic system namely non-ribosomal peptide synthetases, terpene synthases and polyketide synthases for construction of the main skeleton of non-ribosomal peptides, terpenoids and polyketides, respectively. In addition, the cluster includes the tailoring enzymes-encoding genes that modify the skeleton of secondary metabolites (oxidoreductases, acyltransferases, glycosyltransferases and methyltransferases) [147]. Mining of genomes of *Aspergillus* spp., revealed the existence of about 40 cryptic biosynthetic gene clusters for secondary metabolites per genome [136], which are silent under standard laboratory conditions [148]. The successful approaches to activate the gene clusters could be via overexpression of transcriptional factors, promoter exchange and other pleiotropic regulators, epigenetic modulation and simulation of the natural habitat of the same ecosystem will promote the activation of silent gene clusters.

With the revolution of high-throughput genome sequencing and assembly, combined with the development of novel bioinformatics pipelines for identification of gene clusters, scientist noticed the unexplored potential for novel metabolites production are hidden in microbial genomes [95,147,149,150,151,152]. Genome mining for predicting and isolating natural products based on genetic information without experimental trials, has been inspired by the observation that each microbial strain contains the molecular potential to synthesize much more compounds than experimentally detected. So, these computational tools provide a culture-independent route to find new secondary metabolites where traditional laboratory-based approaches fail. Thus, integrating computational and experimental technologies together into a comparative platform that can address large-scale natural product characterization projects will enable further exploration of the natural products [153]. With the development of high-throughput sequencing and computational analysis, genome sequence-based mining approaches could be summarized according to their evolutionary records in the following; (1) Classical genome mining, (2) Comparative genome mining, (3) Phylogeny based genome mining, (4) Resistance/target based genome mining, (5) Metagenome mining, (6) single cell genome mining [154].

### 8.1. Classical Genome Mining

Mining of genes encoding enzymes putatively essential in biosynthesis of target secondary metabolite, is the most “classical” variant of genome mining. Strikingly, with the diversity of secondary metabolites, the biosynthetic machineries of many of these compounds are conserved, as shown from the similarity of amino acids sequence of active site domains of the core biosynthetic enzymes. These core enzymes essential for synthesis of the skeleton of secondary metabolites are polyketides synthase (PKS), non-ribosomal peptides synthase (NRPS) and terpenoid synthase [3,13]. As well as, these conserved genes of characterized pathways were labeled and used as probes in Southern hybridization experiments [154]. The primers deduced based on the highly conserved motifs of these signature genes, were used for PCR screening approaches [3,21,24,27,28,61,78,97,99,103].

Ascertaining the presence of key genes encoding the rate-limiting enzymes of the target biosynthetic pathway could serve as a marker for the potentiality of these endophytes to produce those metabolites [98,155]. For example, taxol producing potency of endophytic fungi could be assessed by the PCR screening of the rate-limiting enzymes of Taxol biosynthesis. It has been reported that fungi showing amplification of DNA fragments specific to genes involved in taxol biosynthesis taxa-4(5),11(12)-dienesynthase (*ts*), dibenzoyl-taxane-2′-a-*O*-benzyoltransferase (*dbat*) and baccatin III13-*O*-(3-amino-3-phenylpropanoyl) transferase (*bapt*) [13,33,34,59]. For this purpose, several software package such as BLAST, DIAMOND, BAGEL, CLUSEAN are usually used [156,157,158] as Secondary Metabolite Bioinformatics Portal (http://www.secondarymetabolites.org). However, the most common computational software packages antiSMASH (antibiotics and Secondary Metabolite Analysis SHell) has emerged as a popular tool for prediction of the biosynthetic gene cluster [159,160,161]. Genome mining of tailoring enzymes, that are involved in modifying precursor molecules, can be valuable targets to identify new BGCs, in addition to the core biosynthetic enzymes [162], as extensively reviewed by [154].

### 8.2. Comparative Genome Mining

The comparative genome mining strategy based on refining both single core biosynthetic genes and partial/ complete gene clusters by the software package antiSMASH, which can compare the identified BGCs of the target genome with the huge collection of BGCs of other microorganisms and the curated MIBiG database [161]. Practically, with the effectiveness of targeted genome mining approaches in identification and prediction of new compounds and their interactions of biosynthetic pathways, however, this approach requires sequences of known homologous enzymes. Another approach to find unknown types of biosynthetic gene clusters in filamentous fungi, is to use algorithm to identify homologous and orthologous gene clusters in related species.

### 8.3. Resistance/target Genome Mining

Resistance based target mining approaches are the recently developed genome mining ones for detecting secondary metabolite gene clusters based on the self-resistant mechanisms of antimicrobial producing organism [154]. The technique is based on the concept that gene clusters contain the biochemical enzymes for compound production, regulatory elements, transporter proteins and resistance mechanisms. The microbial cells that produces a bioactive compound needs to develop self-resistant mechanisms in order to avoid suicide [163]. The resistant mechanisms vary and include efflux pumps, degrading enzymes to remove toxic compounds and modified target proteins to prevent binding of antibiotics to the active site of their targets [164]. For example, the gene clusters responsible for the biosynthesis of novobiocin, platensin and griselimycins [165], containing a second copies of resistant housekeeping genes (gyrB, fabB/F and dnaN) are directly encoded within the gene cluster [154]. So, correlating the putative resistance genes with the target orphan secondary metabolite gene clusters could be an efficient way to mine microbial genomes specifically for antimicrobial compounds.

## 9. Conclusion and Future Directions

Taxol is the most effective anticancer drug with highest annual sale, since its discovery in 1971, however, the lower yield of Taxol from its natural source “bark of *Taxus brevifolia*” and vulnerability of this plant to unpredicted fluctuation with the ecological conditions are the challenges. Endophytic fungi raise the hope for Taxol production due to their fast growth, cost effectiveness, independence on climatic changes, feasibility of genetic manipulation, however, the anticipation of endophytic fungi for this purpose has been halted by the loss of Taxol productivity with subculturing and storage. Thus, this review extensively described the different approaches for Taxol production with emphasizing on usage of endophytic fungi as a potential novel industrial platform for this purpose. In addition, extensive listing of almost of Taxol producing fungal endophytes, different possible approaches to manipulate their Taxol yield such as fermentation bioprocessing, chemical modulation by various elicitors and blockers of competitive pathways, intimate microbiome interaction and metabolic engineering of target pathways have been reviewed. As well as, the metabolic attenuation of Taxol biosynthetic machinery, silencing of the gene clusters encoding Taxol by endophytic fungi and their association to downregulation of transcriptional factors. So, the prospective directions of Taxol production should be focused on screening of novel fungal isolates with sustainable Taxol biosynthetic machinery, further molecular manipulation, exploring the molecular signals and/or transcriptional factors regulating the expression of the Taxol biosynthetic gene cluster.

## Figures and Tables

**Table 1:** Taxol producing fungi and their yields.

Family	Fungus	Host	Taxol Yield µg/L Culture	Method of Assay	Reference
**Taxaceae**	*Taxomyces andreanae*	*Taxus brevifolia*	0.05	CIEIA, HPLC	[111]
	*Alternaria alternata*	*Taxus hicksii*	512	HPLC	[34]
	*Pestalothiopsis microspora*	*Taxus walichiana*	2.9	CIEIA	[112]
	*Nodulisporium sylviforme*	*Taxus cuspidata*	450	HPLC	[113]
	*Cladosporium cladosporioides*	*Taxus media*	800	TLC, HPLC	[110]
	*Aspergillus candidus*	*Taxus media*	112	TLC, HPLC	[114]
	*Phomopsis* sp.	*Taxus cuspidata*	418	HPLC, TLC	[115]
	*Fusarium solani*	*Taxus chinensis*	164	HPLC	[116]
	*Mucor rouxianus*	*Taxus chinensis*	30	HPLC	[29]
	*Aspergillus niger*	*Taxus cuspidata*	273	HPLC	[117]
	*Botryodiplodia theobromae*	*Taxus baccata*	280	HPLC, MS	[118]
	*Taxomyces* sp.	*Taxus yunnanensis*	100	HPLC, TLC	[119]
	*Alternaria alternata*	*T. hicksii*	90	HPLC, TLC	[34]
	*Pestalotiopsis microspora*	*Taxodium distichum*	87	HPLC, TLC	[109]
	*Pithomyces* sp.	*Taxus sumatrana*	84	HPLC, TLC	[111]
	*Pestalotiopsis microspora*	*T. wallichiana*	89	HPLC, TLC	[111]
	*Alternaria* sp.	*T. cuspidata*	19	HPLC, TLC	[120]
	*P. microspora*	*T. baccata*	120	HPLC, TLC	
	*Fusarium lateritium*	*T. baccata*	113	HPLC, TLC	
	*Pestalotia bicilia*	*T. baccata*	125	HPLC, TLC	
	*Monochaetia* sp.	*T. baccata*	190	HPLC, TLC	
	*Kitasatospora* sp.	*T. baccata*	120	HPLC, TLC	[20]
	*Penicillium* spp.	*Taxus species*	111	HPLC, TLC	[20]
	*Pestalothiopsis microspora*	*T. wallichiana*	136	HPLC, TLC	[112]
	*Tubercularia* sp.	*T. mairei*	180	HPLC, TLC	[120]
	*Taxomyces* sp.	*T. yunnanensis*	180	HPLC, TLC	[10]
	*Alternaria alternate*	*T. chinensis*	129	HPLC, TLC	[34]
	*Ozonium* sp.	*T. chinensis*	89	HPLC, TLC	[34]
	*Fusarium mairei*	*T. chinensis*	78	HPLC, TLC	[34]
	*Fusarium solani*	*T. celebica*	75	HPLC, TLC	[34]
	*Botryodiplodia theobromae*	*T. baccata*	45	HPLC, TLC	[34]
	*Botrytis sp*	*T. cuspidata*	65	HPLC, TLC	[117]
	*Fusarium arthrosporioides*	*T. cuspidata*	78	HPLC, TLC	[109]
	*Gliocladium* sp.	*T. baccata*	90	HPLC, TLC	[34]
	*Fusarium solani*	*T. chinensis*	98	HPLC, TLC	[116]
	*Mucor rouxianus* sp.	*T. chinensis*	94	HPLC, TLC	[116]
	*Aspergillus niger var taxi*	*T. cuspidata*	91	HPLC, TLC	[121]
	*Phomopsis* sp.	*T. cuspidata*	82	HPLC, TLC	[121]
	*C. cladosporioides*	*T. media*	72	HPLC, TLC	[110]
	*Aspergillus candidus*	*T. media*	73	HPLC, TLC	[110]
	*Phomopsis* sp.	*T. cuspidata*	70	HPLC, TLC	[110]
	*Pithomyces s*	*T. sumatrana*	20	HPLC, TLC	[122]
	*Didymostilbe* sp.	*T. chinensis*	26	HPLC, TLC	[120]
	*Ozonium* sp.,	*T. chinensis*	29	HPLC, TLC	[121]
	*Alternaria alternata,*	*T. chinensis*	30	HPLC, TLC	[123]
	*Botrytis* sp.,	*T. chinensis*	36	HPLC, TLC	
	*Ectostroma* sp.,	*T. chinensis*	90	HPLC, TLC	
**Podocarpaceae**	*Aspergillus terreus 1*	*Podocarpus gracilior*	20	HPLC, TLC	[103]
	*A. terreus 2*	*Podocarpus gracilior*	14	HPLC, TLC	
	*A. terreus 3*	*Podocarpus gracilior*	18	HPLC, TLC	
	*A. flavus 1*	*Podocarpus gracilior*	4.5	HPLC, TLC	
	*A. flavus 2*	*Podocarpus gracilior*	1.8	HPLC, TLC	
	*Penicillium egyptiacum*	*Podocarpus gracilior*	3.6	HPLC, TLC	
	*Aspergillus terreus 1*	*Podocarpus gracilior*	20	HPLC, TLC	
	*A. terreus 2*	*Podocarpus gracilior*	14	HPLC, TLC	
	*Aspergillus fumigatus*	*Podocarpus* sp.	590	HPLC	[124]
**Other plants**	*Phyllosticta dioscorea*	*Hibiscus rosa-sinensis*	298	HPLC, TLC	[115]
	*Phoma betae*	*Ginkgo biloba*	795	HPLC	[115]
	*Phomopsis sp*	*Ginkgo biloba*	372	HPLC, MS	
	*Phomopsis* sp.	*Larix leptolepis*	334	HPLC, NMR	
	*Penicillium aurantiogriseum*	*Corylus avellana*	70	LCMS, NMR	[125]
	*Bartalinia robillardoides*	*Aegle mamelos*	188	HPLC, MS	[125]
	*Phomopsis* sp.	*Wollemia nobili s*	170	HPLC, TLC	[77]
	*Lasiodiplodia theobromae*	*Morinda citrifolia*	120	HPLC, TLC	[34]
	*Phyllostica melochiae*	*Melochia corchorifolia*	478	HPLC, TLC	[115]
	*Phyllosticta spinarum*	*Cupressus* sp.	235	HPLC, TLC	
	*Phyllosticta citricarpa*	*Citrus media*	265	HPLC, TLC	
	*Fusarium proliferatum*	*Tillandsia usneoides*	165	HPLC	[34]
	*Pestalotiopsis* sp.107	*Tillandsia usneoides*	89	HPLC	
	*Phomopsis* sp. 116	*Tillandsia usneoides*	22	HPLC	
	*Pestalotiopsis* sp., 118	*Tillandsia usneoides*	8.9	HPLC	
	*Pestalotiopsis humus* 133	*Tillandsia usneoides*	6.1	HPLC	
	*Pestalotiopsis humus* 154	*Tillandsia usneoides*	5.7	HPLC	
	*Pestalotiopsis* sp.155	*Tillandsia usneoides*	4.3	HPLC	
	*Pestalotiopsis* sp.163	*Tillandsia usneoides*	4.0	HPLC	
**Rhizosphere**	*Aspergillus flavipes*	Rhizosphere	850	HPLC, TLC	[34]
	*Aspergillus flavus*	Rhizosphere	2.8	HPLC, TLC	
	*Aspergillus oryzae*	Rhizosphere	3.2	HPLC, TLC	
	*Alternaria* sp.	Rhizosphere	4.2	HPLC, TLC	
	*Penicillium chrysogenum*	Rhizosphere	85	HPLC, TLC	
	*Pestalotiopsis malicola*	Rhizosphere	186	HPLC, LCMS	[126]

**Table 2:** Examples of strain fungal improvement approach and fungal endophyte strains with enhanced Taxol production.

Improvement Approach	Wild-Type Strain	Method	Taxol Increasing (Folds)	Reference
**Mutagenesis and molecular manipulation**	*Nodulisporium sylviforme*	UV, EMS, 60Co, NTG	2.5	[121]
	*Fusarium maire*	UV + DES	8.6	[34]
	*Nodulisporium sylviforme*	Genome shuffling	0.5	[113]
	*Ozonium* sp.	PEG-transformation	5	[34]
	*Ozonium* sp.	ATMT	6	[121]
	*Ozonium* sp.	ATMT	N.A	[120]
	*Cladosporium cladosporioides*	ATMT	N.A	[117]
**Cultural nutritional optimization**	*Fusarium mairei*	pH, temperature, carbon, nitrogen source, fermentation period (Single factor)	10.2	[121]
	*F. maire*	Nitrogen source (Plackett Burman design)	1.3	[121]
	*Nodulisporium sylviforme*	pH, temperature, fermentation period (Single factor)	1.15	[113]
	*Pestalotiopsis microspora*	Monobasic sodium phosphate (Single factor)	2.2	[107]
	*Aspergillus terreus*			
**Elicitation/Inhibition Strategy**	*Nodulisporium sylviforme*	Serine, SA, silver nitrate, ammonium acetate	1.1	[113]
	*Periconia* sp.	Serinol, p-hydroxy benzoic acid, β-resorcyclic acid, gallic acid, Benzoic acid	8	[107]
	*Periconia* sp.	Benzoate	8	[121]
	*Fusarium maire*	Sodium acetate	11	[121]
	*Epicoccum nigrum*	Serine	29	[121]
	*Pestalotiopsis microspora*	Fluconazole	50	[107]
	*Aspergillus flavipes*	Fluconazole	50	[34]
**Co-cultivation/mixed fermentation**	*Paraconiothyrium* sp.	*Alternaria* sp.	2.7	[134]
		*Phomopsis* sp.	3.8	
		*Alternaria* sp. and *Phomopsis* sp.	7.8	
	*Fusarium* sp.	Taxus suspension cells	38	[135]
	*Aspergillus terreus*	surface sterilized leaves of *P. gracilior*	2.5	[102]

Ultraviolet (UV), ethyl methyl sulfonate (EMS), nitrosoguanidine (NTG), diethyl sulfate (DES), Agrobacterium tumefaciens-mediated genetic transformation (ATMT).

## Supplementary Materials

The following are available online at https://www.mdpi.com/1420-3049/25/13/3000/s1, Figure S1: Microtubule formation and action of Taxol.
