# Supplementary material for: Exploiting the Biosynthetic Potency of Taxol from Fungal Endophytes of Conifers Plants; Genome Mining and Metabolic Manipulation
Source: Molecules. 2020 Jun 30;25(13):3000. doi: 10.3390/molecules25133000 (PMC7412027; doi:10.3390/molecules25133000)
Supplement: Supplementary file 1 [file molecules-25-03000-s001.pdf]

Supplementari Materials

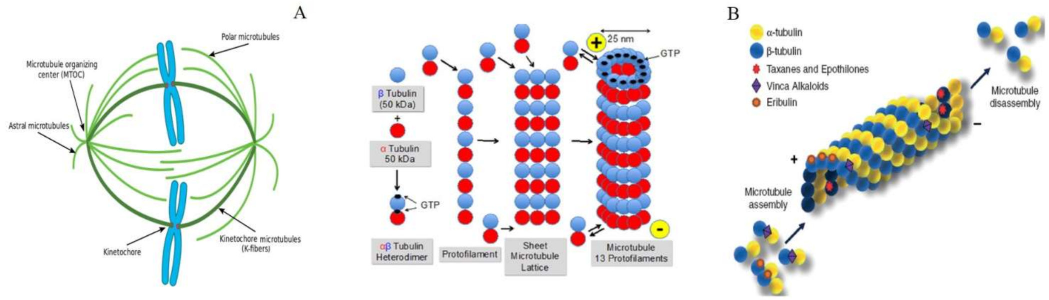

Figure S1. Microtubule formation and action of Taxol. Cellular division and microtubule formation by assembly of α- and β-tubulin via α,β- heterodimers (A), binding to Taxol with the β-tubulin (positive charge) of microtubule resulting in its disassembly (B). http://www.demosmedical.com/
